# Sustainable Composite Materials Based on Carnauba Wax and Montmorillonite Nanoclay for Energy Storage

**DOI:** 10.3390/ma17091978

**Published:** 2024-04-24

**Authors:** Serhii Brychka, Alla Brychka, Niklas Hedin, Mihail Mondeshki

**Affiliations:** 1 Department of Chemistry, Johannes Gutenberg University, Duesbergweg 10-14, 55128 Mainz, Germany; alla.brychka@mmk.su.se; 2 Department of Materials and Environmental Chemistry, Stockholm University, SE-106 91 Stockholm, Sweden; niklas.hedin@mmk.su.se; 3 The Gas Institute of the National Academy of Sciences of Ukraine, 39, Dehtyarivska Str., 03113 Kyiv, Ukraine; 4 Chuiko Institute of Surface Chemistry, National Academy of Sciences, 17 General Naumov Street, 03164 Kyiv, Ukraine

**Keywords:** energy storage, sustainable composite materials, montmorillonite, carnauba wax, NMR spectroscopy

## Abstract

Sustainable composite materials, including carnauba wax, can store energy in the form of latent heat, and containing the wax may allow form-stable melting and crystallization cycles to be performed. Here, it is shown that carnauba wax in the molten state and the abundant nanoclay montmorillonite form stable composites with mass ratios of 50–70% (*w*/*w*). Transmission electron microscopy analysis reveals the inhomogeneous distribution of the nanoclay in the wax matrix. Analyses with infrared and multinuclear solid-state nuclear magnetic resonance (NMR) spectroscopy prove the chemical inertness of the composite materials during preparation. No new phases are formed according to studies with powder X-ray diffraction. The addition of the nanoclay increases the thermal conductivity and prevents the leakage of the phase change material, as well as reducing the time intervals of the cycle of accumulation and the return of heat. The latent heat increases in the row 69.5 ± 3.7 J/g, 95.0 ± 2.5 J/g, and 107.9 ± 1.7 J/g for the composite materials containing resp. 50%, 60% and 70% carnauba wax. Analysis of temperature-dependent ^13^C cross-polarization solid-state NMR spectra reveal the enhanced amorphization and altered molecular dynamics of the carnauba wax constituents in the composite materials. The amorphization also defines changes in the thermal transport mechanism in the composites compared to pure wax at elevated temperatures.

## 1. Introduction

Sustainable composite materials [1] have wide areas of application, including biomedicine [2], tissue engineering [3], packaging [4,5], batteries [6], tires [7], life cycle analysis [8], and construction [9]. Energy storage has an important place among these applications due to the ever-growing need for sustainable energy solutions [10] to reduce the rate of global warming. Energy can be stored in various ways [11], including in the so-called phase-change materials (PCMs), which accumulate latent heat [12]. Numerous recent reviews summarize the types of PCMs, the most common thermal storage methods and materials, as well as the benefits and drawbacks of the different PCMs [13,14,15,16,17,18,19,20,21,22,23,24,25]. A broad range of inorganic and organic PCMs have been studied, and salt hydrates, salt solutions, and compositions with paraffin have been commercialized [12]. The most important implementation of PCMs is in the construction industry for active and passive building applications for s reduction in electrical energy and fuel consumption [23]. It has been shown that, for example, a reduction of 24% in thermal conductivity was achieved, and the specific heat capacity was increased by 17% with the incorporation of PCMs in the construction material [26]. Waxes are one the most important organic PCMs; the most widely studied is paraffin (petroleum) wax due to its high latent heat capacity, satisfactory thermal and chemical stability, no supercooling during phase transition, non-toxicity and non-corrosive quality to metal containers [27]. Its melting temperature range of 60–75 °C is convenient for heat storage applications [28]. However, paraffins are produced from non-renewable sources, and their carbon footprint and associated environmental impact cannot be overlooked [29]. In addition, paraffin wax has a low thermal conductivity, hindering its heat charging and discharging process. This problem can be circumvented using small amounts of carbon nanotubes [30] or graphene [31] as additives. PCMs based on paraffin wax with aluminum-based additives like α-nanoalumina have also been studied [32]. The addition of 8% aluminum foil in paraffin wax has been shown to double the thermal conductivity to reach values of 0.63 W/Mk [33]. Also, additions of expanded graphite have been shown to enhance the thermal conductivity by more than 200% at a loading of only 10% [34]. Other compositions have been shown to increase the thermal conductivity and specific heat capacity [35]. Waxes from nature are alternatives to paraffin waxes in many applications [36,37,38]. Here, it can be noted that the shellac wax has comparable heat storage efficiency and could be a potential replacement for paraffin wax when it comes to solar thermal applications, including domestic water heating and food drying in the temperature range of 60–80 °C [39]. Sugar cane wax-based PCMs have also been studied [40].

Carnauba wax (also called Brazil or palm wax) is a renewable, cost-effective material produced in large amounts from the leaves of the carnauba palm *Copernicia prunifera*. Its melting point is above 80 °C, which may lead to a significant increase in the heat capacity of composite materials [41]. The production process includes collecting and drying the leaves, beating them to loosen the wax, then refining and bleaching the wax [42]. Carnauba wax consists mostly of aliphatic esters (≈40%), diesters of 4-hydroxycinnamic acid (≈21%), ω-hydroxycarboxylic acids (≈13%), and fatty alcohols (≈12%). The ingredients are predominantly derived from acids and alcohols in the C_26_–C_30_ range. Other (unsaturated) components are also present, however, in lower concentrations [43].

Developing mixed organic PCMs with clays and waxes from nature seems to meet the criteria of “green” chemistry [10] and facilitates the creation of dimensionally stable composites [44]. The clay mineral materials that are the most important, including kaolin, diatomite, sepiolite, montmorillonite, perlite, SiO_2_, attapulgite, vermiculite, and fly ash, have relatively high thermal conductivity and excellent absorbability [44] which effectively prevents the leakage of the (typically) organic PCMs [45]. Other advantages include high thermal stability and low costs of production. Montmorillonite is a layered mineral built of two tetrahedral sheets of silica sandwiching an alumina-based central octahedral sheet. The clay particles are plate-shaped with an average diameter of ca. 1 μm and a thickness of 0.96 nm. As a member of the smectic group, it comprises a range of smectites differing mainly by the structure of the octahedral sheet. This is expressed in the general formula as follows: $M_{x/\vartheta }^{\vartheta +}\left({Si}_{4-y}{Al}_{y}\right)[{\left(Al{,Fe}^{3+}\right)}_{2-z}({Mg,{Fe}^{2+})}_{z}]O_{10}{\left(OH\right)}_{2}$, where x = ξ = 0.2–0.6, x = y + z, and y << z. $M^{\vartheta +}$ represents the interlayer cations such as Na^+^, K^+^, Ca^2+^, or Mg^2+^ and ξ is the total charge [46]. The substitution of lower valence cations leaves the nearby oxygen atoms with a net negative charge that can attract cations [47]. Beidellitic substitution related to the tetrahedral silica sites also occurs. The charge location allows montmorillonite to be defined as having a >10% tetrahedral charge, while minerals with ξ in the range of an 11–50% charge and associated with the tetrahedral sites are classified as beidellitic montmorillonites [46].

Depending on the fabrication method, composite materials of organics and clays can be prepared as physical mixtures [48] or as hybrid materials where the interpenetration of the aliphatic chains in the interlayer spacing can take place [49,50]. In all cases, phase-stable composite materials with high heat capacity and durability, in which the montmorillonite enhances the heat transfer, are formed [51,52]. Not only liquid–solid but also solid–solid montmorillonite-based composite materials where no leakage can take place are synthesized. Organic montmorillonite supports crosslinking and improves on the temperature control capacity, the crystallization ability, and the thermal stability of the solid–solid phase change polyurethane material [53]. Also, the phase-change enthalpies of reduced graphene oxide–montmorillonite-poly(vinyl alcohol) PCM composites were high, and the composites’ thermal cycle stability was over 2000 thermal cycles [54]. The thermal conductivity of the PCM composites was enhanced in comparison with pure poly(vinyl alcohol), and the sunlight absorbability increased from 40 to 90% upon loading with a 5% content of graphene oxide. In the case of capric–stearic acid/montmorillonite/graphene composites, it was shown that the montmorillonite prevents the leakage of the PCMs while the thermal conductivity increases with the small addition of graphene [55]. Composite PCMs based on organic montmorillonite/paraffin/grafted multi-walled nanotubes have been studied; the latent heat was 47.1 J/g, and the thermal conductivity of the PCM composites was higher than that of paraffin [56]. In another study, the montmorillonite framework was exfoliated into two-dimensional montmorillonite nanosheets that allowed the encapsulation of 95% stearic acid without leakage, resulting in the highest latent heat capacity of 199 J/g among clay mineral-based composite PCMs. The composite also had rapid heat transfer paths, an outstanding thermal transfer ability, and excellent photo-thermal conversion performances [57]. Under acidic conditions, montmorillonite is transformed into halloysite, which is an oxide clay silicate mineral that is abundantly available worldwide. The cavities of halloysite nanotubes have been filled with various molecules to create effective and functional materials [41,58,59].

Knowledge about the first-order phase transitions and the latent heat of PCMs, as well as the chemical stability of the PCMs over thermal cycling [60,61], is important when designing heat storage systems. Numerous methods give physicochemical insights into the changes related to phase transitions, including solid-state nuclear magnetic resonance (NMR) spectroscopy [62]. The method provides information based on the electronic environment of magnetically active nuclei, which facilitates studying not only organic and inorganic phase change materials and composites but also crystalline and amorphous systems. Changes in NMR relaxation times [63,64], spectral parameters [65], as well as translational [66] and spin diffusion [67], can be studied and analyzed. The molecular structure of beeswax has been revealed using ssNMR spectroscopy [62,68].

In this contribution, we significantly extend an earlier report [69] on sustainable composite materials based on mixtures of the carnauba wax and the abundant and cost-effective montmorillonite nanoclay. Thermal properties are studied. It is also shown that the nanoclay particles are non-uniformly distributed and that the system is chemically inert. Changes in the crystalline and amorphous states are studied. Furthermore, variable temperature (VT) ^13^C cross-polarization (CP) NMR spectra imply a change in the thermal transport mechanism, increasing the temperature in the solid phase (below the wax melting point).

## 2. Materials and Methods

Montmorillonite K10 nanoclay (CAS No: 1318-93-0; surface area according to the quality certification is 220–270 m^2^/g) was purchased from Sigma Aldrich Chemie GmbH (Steinheim, Germany). Carnauba wax (CAS No: 8015-86-9; it is in the form of yellow-brown flakes) was purchased from Naturprodukte Lembke GbR (Faulenrost, Germany).

Montmorillonite was mixed with carnauba wax at a temperature of 120–130 °C using a magnetic stirrer for 30 min before it was rapidly cooled with cold water to prevent the sedimentation of the nanoclay. The obtained solid samples of composite materials of wax/nanoclay with 50/50, 60/40, and 70/30 mass ratios were ground for 20 min to provide polydisperse powders, which were used for all experiments [69]. Further heat treatment (annealing) at 60 °C for 1 month of a sample of PCMs of wax/nanoclay and a 50/50 mass ratio was applied to study the effect of temperature on the storage condition of PCMs.

### 2.1. Differential Scanning Calorimetry (DSC)

Differential scanning calorimetry (DSC) curves were recorded using a TA Instruments Discovery DSC 250 (TA Instruments, New Castle, DE, USA) equipped with a 50-position autosampler and a TA Refrigerated Cooling System 90 (New Castle, DE, USA), applying indium and n-octane as calibration standards. The instrumental temperature accuracy is ±0.025 °C, and the calorimetric accuracy is 0.25%. For each sample, two heating and one cooling cycle were conducted at linear heating rates of 10 °C/min under a nitrogen atmosphere in a temperature range of 0 to 200 °C. The samples of polydisperse powder weighing about 10 mg were placed in a sealed pan. The recording of the DSC curves began at 25 °C. The DSC analyses were performed using the Trios (version 5.5.1.5) software, integrating the region between 30 °C and 100 °C. Normalization was performed by dividing the heat flow by the sample amount.

### 2.2. Powder X-ray Diffraction (PXRD)

The samples for the PXRD measurement were prepared by grinding the powders in an agate mortar and subsequent bulk formation. Powder X-ray diffraction (PXRD) was conducted at ambient conditions using a Bruker D8 DISCOVER diffractometer (Bruker, Karlsruhe, Germany) with a wavelength of α_1_ = 1.5406 Å and α_2_ = 1.5444 Å of Cu*K_α_*-radiation with the detector LYNXEYE XE-T in the configuration of Bragg–Brentano. The spectra were recorded in the range of angles 2θ = 5–90° with a rotation speed of 0.02°·min^−1^.

### 2.3. Infrared (IR) Spectroscopy

IR spectroscopy was performed at room temperature on a Varian 670-IR spectrometer (Varian, Mulgrave, Australia) equipped with a single reflection ATR device (Specac, Orpington, UK) with a diamond ATR element in the range of 4000–390 cm^−1^ with a resolution of ±2 cm^−1^.

### 2.4. Transmission Electron Microscopy (TEM)

Samples were prepared by drop-casting the respective sample dispersion on 200 mesh formvar carbon copper grids and measured on a JEOL JEM-2100F microscope (Jeol, Tokyo, Japan) with an acceleration voltage of 25 kV.

### 2.5. Scanning Electron Microscopy (SEM)

The SEM images were obtained with a JEOL JSM IT800 Schottky Field Emission scanning electron microscope (Jeol, Tokyo, Japan) in the backscattered electron images mode in a low vacuum.

### 2.6. Solid-State NMR Spectroscopy

All solid-state (SS) NMR spectra were recorded on a Bruker Avance 400 DSX NMR spectrometer (Bruker BioSpin GmbH, Rheinstteten, Germany, operated by Topspin 1.3, 2007, patch level 8) at a ^1^H frequency of 399.87 MHz, ^23^Na frequency of 105.77 MHz, ^27^Al frequency of 104.19 MHz, ^13^C frequency of 100.55 MHz and ^29^Si frequency of 79.44 MHz. A commercial three-channel and 4 mm Bruker probe head was used for all experiments. A 10 kHz magic angle spinning (MAS) rate was used. The variable temperature (VT) ^1^H NMR spectra were recorded averaging 32 transients with a 5 s recycle delay. The temperature correction due to the frictional effect of the bearing gas was performed following a known procedure [70]. For all solid-state ^13^C CP MAS NMR experiments, an initial 90° pulse with a 4.0 μs length and a 4 s recycle delay were used. A ramped CP pulse (from 64 to 100%) with a duration of 2 ms was used to record the VT spectra. A two-pulse phase modulation (TPPM) ^1^H decoupling scheme [71] was used while acquiring the ^13^C signal. In total, 20 k transients were averaged for the CP experiments. The spectra were baseline-corrected, and a broadening of 30 Hz was applied. The spectra were referenced to external adamantane at 1.63 ppm (^1^H) and 38.5 ppm (^13^C). The ^23^Na NMR spectra were recorded using a single pulse excitation scheme averaging 4 k scans with a 5 s recycle delay. The spectra were baseline-corrected, and a broadening of 150 Hz was applied before the Fourier transformation. The ^23^Na spectra were referred to the ^23^Na resonance at 7.2 ppm in crystalline NaCl as a secondary reference. The ^27^Al NMR spectra were recorded using a short flip angle excitation (equal to π/12), averaging 512 scans with a 2 s recycle delay. A broadening of 100 Hz was applied before processing the spectra. Additionally, the ^1^H-^27^Al CP NMR spectrum with a 2 ms contact time was recorded using an initial 90° pulse with a 4.0 μs length, 3 s recycle delay, and averaging 3 k scans under ^1^H TPPM decoupling. The ^27^Al spectra were referenced to an external 1 M water solution of AlCl_3_ at 0 ppm as a secondary reference. The ^1^H-^29^Si CP NMR spectra were recorded using an initial 90° pulse with a 4.0 μs length, 3 s recycle delay, and averaging 15 k or 50 k scans under proton TPPM decoupling. The contact time used was 8 ms. The ^29^Si spectra were referenced to external solid TTSS (tetrakis-(trimethylsilyl)-silane) at −9.9 ppm as a secondary reference.

Data from *PXRD*, *FTIR,* and *DSC* were plotted using Origin 2020 (OriginLab Corporation, Northampton, MA, USA).

## 3. Results and Discussion

**Figure 1:**
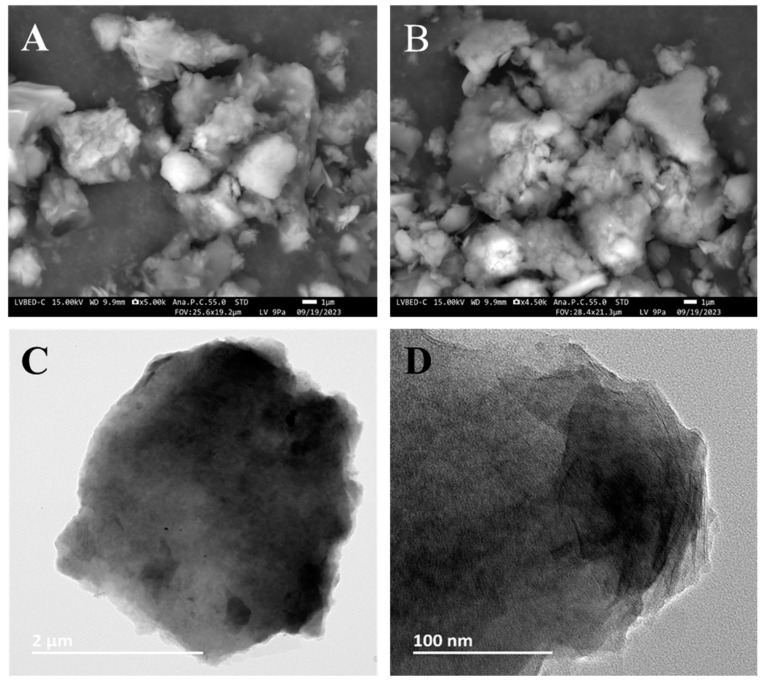
(**A**,**B**). SEM micrographs of a montmorillonite and carnauba sample (MCS_50:50_, (*w*/*w*)). (**C**,**D**). TEM images of MCS_50:50_ obtained at different magnifications.

Montmorillonite and carnauba wax were melt-mixed and rapidly cooled down, and ground, and montmorillonite and carnauba samples (MCS_X:Y_) with mass ratios of 50/50, 60/40, and 70/30 were prepared. The SEM images of MCS_50:50_ delivered information on particle agglomerates of about 10–30 µm in size and indicated that the sample had a relatively large surface area (see Figure 1A,B). The TEM images of the same MCS_50:50_ in Figure 1C,D provide information on the clay nanoparticles in the composite nanomaterials. These particles can be identified by the difference in contrast since they are darker than the organic matrix. Some domains of the composite materials are dominated by the wax matrix while others are richer in nanoclay. The consequences of this inhomogeneity are discussed in terms of solid-state NMR results.

**Figure 2:**
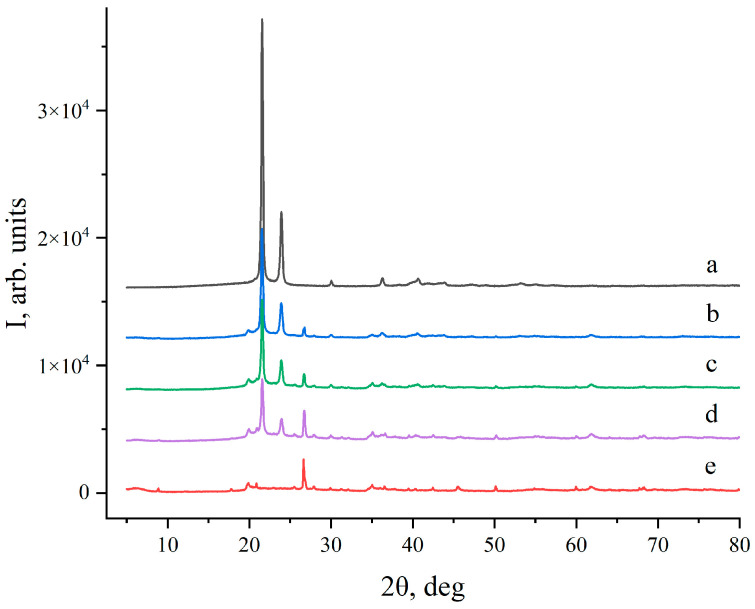
Powder XRD patterns of pure wax (a), composite montmorillonite and carnauba samples MCS_70:30_ (b), MCS_60:40_ (c), MCS_50:50_ (d), and pure montmorillonite clay (e).

The carnauba wax is arguably the most important wax in nature when it comes to applications and economics [72]. It is extracted from the Brazilian palm *Copernicia prunifera*. Its crystals have an orthorhombic crystal structure (a ≠ b ≠ c) [73] characterized by two intense maxima at 2θ = 21.55° (110) and 23.9° (200), as can be observed in the X-ray diffractogram in Figure 2(a). Waxes that crystalize in orthorhombic structures are characterized by the low freedom of rotation of the methyl groups [59] as well as the higher hardness and lower permeability of foreign gas and other impurities. They are also less prone to deformation than waxes that crystallize in hexagonal structures.

The synthesis of the composite materials does not lead to any significant changes in the position and intensity of the reflections related to both wax and clay (Figure 2). The montmorillonite (001) reflection (2θ = 8.86°) remains unaffected in the composites, as can be deduced by comparing the diffractogram of the pure clay (Figure 2(e)) with those of the MCSs (Figure S1). This similarity excludes (significant) the interlayer penetration of the wax components into the clay layers. The most intense (001) reflection in the montmorillonite diffractogram originates from the interlayer distance in the single crystals. This reflection could broaden and shift towards small angles, providing the interlayer intercalation of organic wax components during the synthetic procedure [74]. Impurities of quartz and feldspar are also present in the nanoclay (Figure S2). Carnauba wax reflections also retain, in a consistent manner, their positions in the diffractogram of the MCSs. Furthermore, no reflections related to new phases were observed. Thus, according to the X-ray analysis, the prepared MCSs are physical mixtures of carnauba wax and montmorillonite nanoclay. This also points towards chemical stability (at the synthetic conditions), which is a requirement for materials used for the accumulation of thermal energy [75]. It should also be noted that the recorded diffractograms do not reveal (significant) amorphous regions in the pure inorganic and esp. in the organic phases as well as the composite materials. Such regions can be studied by solid-state NMR spectroscopy as it relies on (differences in) the electronic environment of the NMR active nuclei and not on the crystalline order.

**Figure 3:**
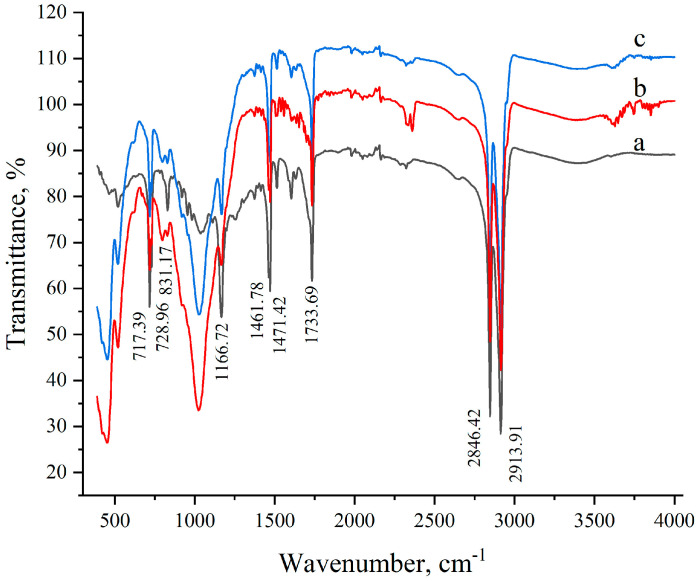
IR spectra of the pure wax (a), pristine montmorillonite and carnauba sample (MCS_50:50_, (*w*/*w*)) (b), and MCS_50:50_ cycled 500 times in a heating–cooling cycle (c).

The chemical stability (at synthetic conditions) of the composite material components was investigated by the analyses of ATR-IR and multinuclear solid-state NMR spectra. The ATR FTIR spectrum of carnauba wax is presented in Figure 3. The absorption maximum at ca. 3400 cm^−1^ is due to the O–H stretching vibration of the hydroxyl groups of fatty alcohols present in the wax. The spectrum is dominated by the absorption bands related to the abundant methylene groups. The absorption lines at 2916 cm^−1^ and 2848 cm^−1^ are associated with the C-H asymmetric and symmetric in-plane stretching vibrations, respectively, while the 1462 cm^−1^ absorption corresponds to the scissor’s deformation vibrations in the CH_2_ groups. The absorption bands at ca. 717–729 cm^−1^ are due to deformation rocking vibration in the CH_2_ in differently oriented crystalline chains, which perform the same motion, however, with a phase difference [76]. Absorptions related to methyl groups are also detected; however, not so intense due to the smaller number of the methyl groups in the wax mixture. The asymmetric in-plane CH_3_ vibration is observed as a shoulder at 2954 cm^−1^, while the line related to the symmetric in-plane vibration (expected to appear at ca. 2870 cm^−1^) overlaps with the one for the CH_2_ groups. The CH_3_ line related to the deformation out-of-phase bending vibration overlaps with the one for the CH_2_ groups, while the in-phase deformation is detected at 1378 cm^−1^. The strong absorption band at 1734 cm^−1^ is due to the stretching vibration of the carbonyl group C=O of the (≈40%) aliphatic esters [76]. In this spectral region, the C=O group of the diesters of 4-hydroxycinnamic acid are also absorbed (≈21%). The lower intensity broadened shoulder shifted to higher frequencies due to dimers of the ω-hydroxycarboxylic acids (≈13%) as well as hydrogen-bonded acids and fatty alcohols. The strong band at 1167 cm^−1^ of the C–O stretching vibration of the ester is also detected. The ATR IR spectrum of montmorillonite is presented in Figure S3. Two absorption maxima in the high-frequency range are observed at ca. 3400 cm^−1^ due to the O–H stretching vibration of the structural OH groups and water and at 3618 cm^−1^ for the admixture of quartz and montmorillonite [53,55]. The broadened intense absorption band at 1007 cm^−1^ is associated with the Si–O–Si antisymmetric stretching vibration [48,55], while the shoulder at 950 cm^−1^ is due to the bending in-plane vibrations of the Si–O and Al–O bonds of the tetrahedral silica and octahedral alumina in the structure of the clay layers [50]. The fingerprint region is characterized by a sharp absorption at 521 cm^−1^, representing the Al–O stretching vibration, and at 420 cm^−1^ due to the Si–O–Si bending vibration of the tetrahedral silica layers of montmorillonite [55]. In the IR spectra of the MCSs, all the absorption bands characteristic of the original components were detected. The identical positions of the maxima modes in Figure 2 and Figure S3 proved the absence of changes in the molecular structures caused by chemical reactions. A detailed analysis of the 1660–1640 cm^−1^ IR region revealed no changes in the intensity and position of the maximum for all samples (Figure S3). Therefore, the proposed system meets the requirements of chemical stability at the stage of composite materials preparation.

To investigate the applicability of the synthesized composite materials in the construction industry, 500 heating/cooling cycles were performed on the MCS_50:50_ sample within the temperature range of −20 °C to 80 °C. The IR spectra of the pristine and cycled MCS_50:50_ hardly differ (within the experimental error) from one another (Figure 3(b,c)). The almost identical form of the spectra confirms the chemical stability of the MCS_50:50_ sample and indicates its suitability for application as a PCM.

**Figure 4:**
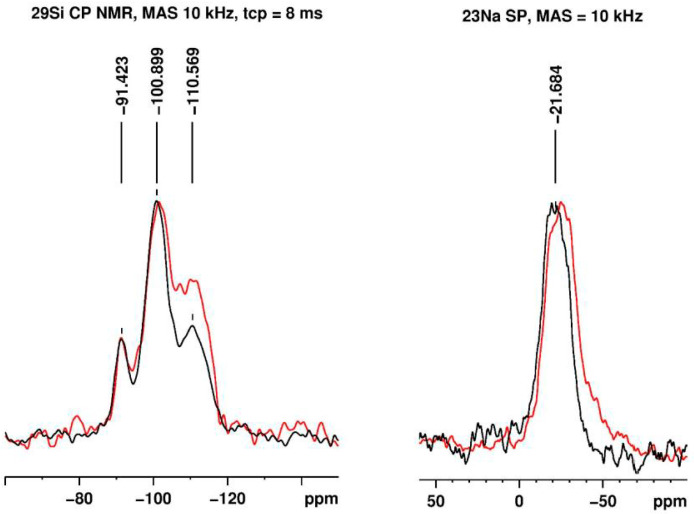
^29^Si cross-polarization and ^23^Na single pulse excitation NMR spectra of the pure montmorillonite (black) and the MCS_50:50_ (red) recorded at 10 kHz MAS. The spectra are scaled to equal intensity.

To further investigate the interaction between nanoclay and carnauba wax, we recorded ^29^Si cross-polarization (CP), ^23^Na single pulse (SP) excitation, and ^27^Al SP and CP NMR spectra. The ^29^Si CP NMR spectra and the ^23^Na SP NMR spectra of pure carnauba wax and MCS_50:50_ are presented in Figure 4.

The ^29^Si CP NMR spectrum of the montmorillonite nanoclay (Figure 4 left black line) displays three resonances at ca. −91 ppm, −100 ppm, and −110 ppm related to the Q^2^, Q^3^, and Q^4^ units of tetrahedrally coordinated silicon, respectively. As expected, no change in the ^29^Si chemical shifts occurred after the nanocomposite synthesis, as well as no new shifts in the spectra of the mixtures, as no chemical reaction involving the silicon atoms (or their immediate environment) took place (Figure 4 left). However, the ^29^Si CP NMR spectra reveal the ratio between the integrals related to the units of change from ca. 0.25:1:0.5 (−91:−100:−110 ppm) for the nanoclay to ca. 0.2:1:0.9 for the composite material (the deconvoluted spectra are presented in Figures S4 and S5 and the NMR parameters are in Tables S1 and S2). The reason for this is that more Q^4^ units in the composite materials are in a through-space contact (dipolar coupled) with protons of the carnauba wax components. This facilitates the magnetization ^1^H-^29^Si transfer during the CP pulse sequence and results in an overall increase in the ^29^Si signals intensity and, in particular, in a stronger relative increase in the Q^4^ integral. Such an increase is a confirmation of a successful composite synthesis.

The ^23^Na SP solid-state NMR spectra (Figure 4 right) of the nanoclay (black line) and the composite material MCS_50:50_ (red line) provide information about the sodium ions incorporated in the montmorillonite-layered structure. The spectra reveal a slight shift of the ^23^Na chemical shift of about 4 ppm to a higher field after the composite material synthesis. This could be related to either an interaction between the carboxy- or ester functional groups of the organic wax components with the sodium ions or to additional shielding achieved after the composite formation. It should be noted that possible (electrostatic) interactions between organic oxygen-containing functional groups and sodium ions can imply short distances between ^1^H and the ^23^Na atoms (either as an intercalation of some alkyl chains between the tetrahedral silica and the octahedral alumina sheets of individual crystals or a diffusion of the sodium ions towards the surface of the crystals). This, on its own, can facilitate ^1^H-^23^Na magnetization transfer in a cross-polarization experiment. The latter experiment (spectrum not presented), however, resulted in no visible ^23^Na resonances. Thus, the 4 ppm high field shift is related to further electronic shielding resulting from the mere presence of the organic wax components around the montmorillonite-layered structure.

The ^27^Al solid-state NMR spectrum of the pure montmorillonite and in the composite material MCS_50:50_ is presented in Figures S6 and S7 (after deconvolution). Three resonances were detected at 3.3 ppm, 56.0 ppm and 70.0 ppm. The most intense signal at 3.3 ppm was related to the symmetric octahedral AlO_6_ site (^VI^Al). Tetrahedrally coordinated aluminum AlO_4_ resonates in the chemical shift range between 40 and 90 ppm. In the case of layered-lattice aluminosilicates, some of the silicon atoms can be replaced by aluminum ones, thus forming defect sites where the ^29^Al shift is in the range of 70–80 ppm [77]. Hence, we assign the signal at 70.0 ppm to aluminum in a defective Q^3^ (3Si) structure. The low-intensity resonance at 56 ppm is probably due to a low amount of tetrahedrally coordinated aluminum in a Q^4^ (4Si) structure. An approximately linear relationship for a number of layer lattice aluminosilicates between the chemical shift of the tetrahedral Al and the tetrahedral composition, expressed as the ratio Si/(Si(IV) + Al(IV), has been earlier reported [46]. The presence of carnauba wax in the composite material did not influence the ^27^Al shifts (Figure S6).

The presented experimental data confirm the conclusion of the IR spectroscopic studies regarding the low chemical activity of the components of the mixture in the process of the composite materials preparation.

We recorded temperature-dependent ^1^H and ^13^C cross-polarization (CP) NMR spectra to evaluate the effect of temperature on the segmental dynamics of pure carnauba wax below the melting point for pristine MCS_50:50_ and annealed MCS_50:50_ (annealed for a month at 60 °C). The variable temperature ^1^H NMR spectra (Figure S8) are severely broadened due to the (net of) homonuclear dipole–dipole couplings, which cannot be averaged by the intermediate 10 kHz spinning at the magic angle to improve the resolution. The ambient temperature spectra of pristine MCS_50:50_ show a maximum at 0.78 ppm; the maximum in annealed MCS_50:50_ is shifted ca. 0.1 ppm to the lower field. This dominant resonance is due to the protons of the aliphatic esters and fatty alcohols in the C_26_–C_30_ range. The changes related to the enhanced dynamics at higher temperatures are detected in the spectra of all samples; however, they are most pronounced in the spectrum of pure wax. In this case, the peak at 0.78 ppm becomes sharper, and a second resonance at 0.42 ppm is additionally detected. The montmorillonite nanoparticles induce the broadening of the ^1^H signals, esp. close to the nanoparticle surface, due to the lower degrees of motional freedom of the organic components (resulting in reduced dynamics). The broad resonance at ca. 7 ppm suggests the presence of aromatic protons, which correlates well with the carnauba wax composition, i.e., containing 4-hydroxycinnamic acid (p-coumaric acid) diesters.

**Figure 5:**
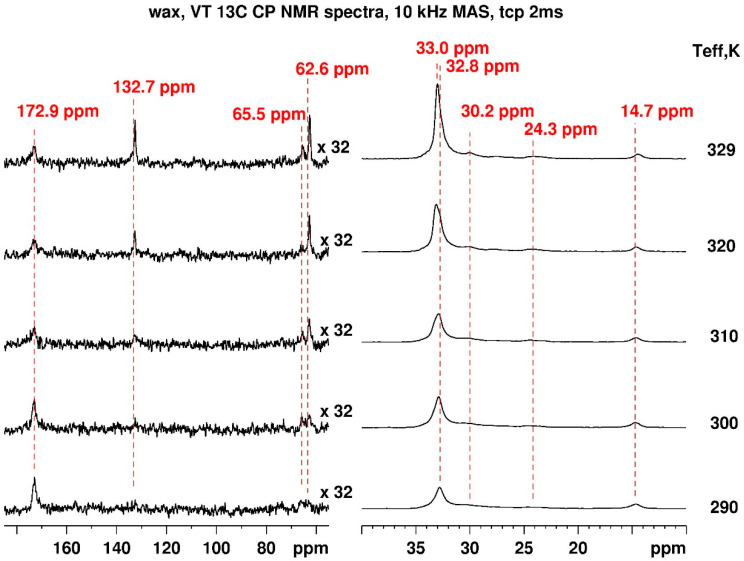
VT ^13^C CP NMR spectra of carnauba wax recorded at 10 kHz MAS with a contact time of 2 ms under proton decoupling with the effective sample temperature presented on the right.

The VT ^13^C CP NMR spectra of the pure carnauba wax, pristine MCS_50:50_, and annealed MCS_50:50_ are presented resp. in Figure 5, Figures S9 and S10. The ^13^C spectrum of the wax is dominated by the resonances of the aliphatic carbons at 14.7 ppm (terminal methyl carbons), 24.3 ppm, 30.2 ppm, and ca 33.0 ppm for the chain methylene carbons and 62.5 and 65.5 ppm for -CH_2_-OH, -OCH_2_- groups. Additionally, the signals at 132.7 ppm and 172.9 ppm are due to aromatic and carboxyl carbons, respectively [78,79]. Below, we discuss the intensity and chemical shift changes in the ^13^C resonances in terms of crystallinity and motional behavior for the pure carnauba wax and then compare this with the mixture of montmorillonite and the mixture after long annealing.

The chemical shift at 30 ppm arises from methylene groups in gauche-containing conformers (comparable to the polyethylene amorphous regions), while the 32.8 ppm methylene peak reveals stretched C_26_–C_30_ chains adopting trans conformation [80]. The predominant trans conformation suggests the presence of abundant crystalline regions in the aliphatic chains with minor amorphous domains. The ratio between the integrals of the resonances increases in favor of the gauche conformation, raising the temperature and ca. 32.8 ppm:30 ppm = 3.85:1 determined at 329 K. A slight shift of about 0.2 ppm in the lower field of the initial 32.8 ppm signal as well as sharper resonances due to enhanced dynamics are detected increasing the temperature (however, below the melting point). The temperature-dependent behavior of the -OCH_2_- and aromatic carbon resonances at 62.6 ppm, 65.5 ppm, and 132.7 ppm is atypical. Higher temperatures result in higher molecular mobility, which leads to reduced heteronuclear ^1^H-^13^C dipolar couplings (required for the cross-polarization transfer) and lower cross-polarization efficiency and signal intensity, respectively. In this case, at higher temperatures, contrary to the expected signal reduction (as for the carboxy carbon at 172.7 ppm), we observed a signal increase. We can attribute the lack of signal for the -OCH_2_- and aromatic carbons (p-coumaric acid diesters) at ambient conditions to the interference of the local dynamics with the magic angle spinning, cross-polarization, and/or dipolar decoupling of the order 10–20 kHz [81].

The presence of the nanoparticles induces signal broadening for the ^13^C alkyl signals in the whole measured temperature range, as can be seen in the spectra recorded for the pristine MCS_50:50_ in Figure S9. The reason for this is the reduced dynamics close to the nanoparticle surface as well as the (partial) loss of the crystalline order. It can be noted that the “interphases” in nanocomposites [82] make them tougher by reducing the dynamics when the organics bridge the nanoparticles [83]. The ratio between the integrals for the trans- and gauche conformation changes in favor of the latter (33 ppm:30 ppm = 1.25:1 at 329 K) due to an increase in the amorphous regions in the sample. It should be noted that some crystalline domains in the composites were retained due to the inhomogeneous distribution of the nanoparticles in the wax matrix, as revealed by TEM. Such sample inhomogeneity can influence the ratio between the integrals of the trans- and gauche conformations. The resonances related to the -OCH_2_- and aromatic carbons (of the p-coumaric acid diesters) are detected in the whole temperature range due to the broad distribution of the correlation time of the molecular motion (particularly for the p-coumaric acid diester). No significant changes were observed for the carboxyl carbon signal.

Surprisingly, the ^13^C CP spectra after long-term annealing for the annealed MCS_50:50_ (Figure S10) reveal a further disorder in the organic phase as the integral of the gauche conformation resonance grows for the sake of the trans one, the effect of which is visible for the higher temperatures. The ratio between the integrals of the signals at 33 ppm:30 ppm is ca. 1:1 (329 K). It should be noted, however, that the measuring time for one spectrum averaging 20k scans is about 22 h at the specified temperatures, which on its own is a kind of annealing at a moderate spinning speed of 10 kHz. With this in mind, we explain the increased disorder in the organic phase at higher temperatures, possibly due to the formation of H-bonding between the surface of the montmorillonite nanoparticles and the -COO^−^ groups of the wax constituent after long-term annealing. The signal loss for aromatic carbons occurs at higher temperatures as the molecular motion is shifted to higher frequencies. This could be related to improved π–π stacking between the abundant p-coumaric acid analogues. Reduced signal intensity is also characteristic for the -COO^−^- resonance at 172.7 ppm and the -OCH_2_- signal in the range 62–66 ppm due to altered molecular dynamics compared to the pure carnauba wax. The thermal exposure at 60 °C for a month for the annealed MCS_50:50_ results in altering the intermolecular interactions (hydrogen bonding, π–π stacking) between the different constituents of the composite materials as well as the reorganization of the system below melting.

**Figure 6:**
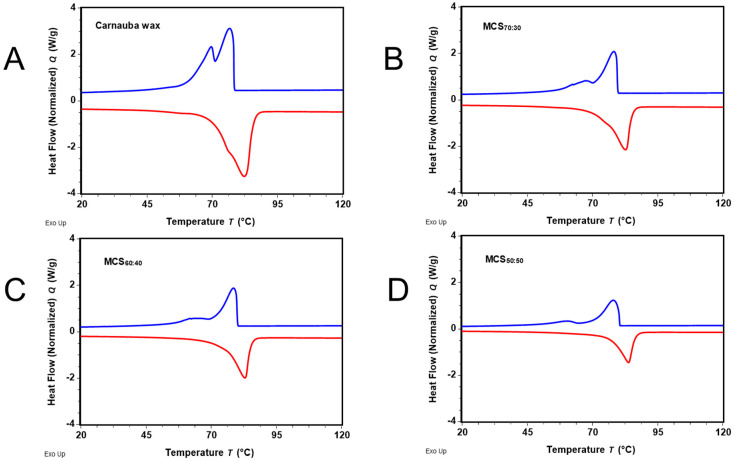
Exemplary DSC curves of heat absorption-desorption of (**A**) pure carnauba wax and its mixtures with montmorillonite clay (*w*/*w*), (**B**) MCS_70:30_, (**C**) MCS_60:40_, and (**D**) MCS_50:50_. Blue and red lines correspond to cooling and second heating respectively.

*Heat accumulation of the composite materials*. PCMs accumulate energy in the form of latent heat in a strictly defined temperature range. The amount of accumulated energy and the respective temperature range could be determined using DSC. The DSC curves were measured by applying a temperature ramp, increasing or decreasing the temperature, and providing information about the melting enthalpy (enthalpy of fusion; endothermic process) and the enthalpy of solidification (exothermic process) as an integral of the DSC peak plotted with respect to the temperature. The DSC curves of carnauba wax and MCSs were recorded in the temperature range of 0–200 °C (Figure 6 and Supporting Information) using a linear heating rate of 10 °C/min. The presented results are an average of three measurements of the inhomogeneous samples. The inaccuracy due to sample inhomogeneity (with the varying composition of the carnauba wax, varying size and morphology of the montmorillonite nanoparticles, and inhomogeneous mixing between both constituents of the composite materials) is significantly higher compared to the instrument precision. The DSC curve related to the cooling cycle of pure wax (Figure 6A) is characterized by two distinct endothermic peaks at ca. 77.1 ± 0.2 °C and 69.9 ± 0.1 °C, which correspond to heat accumulation. Thus, the latent heat of the wax is a sum of the latent heat of different wax constituents. It should be noted that the same phenomenon was also observed for paraffin wax with a melting point in the range of 45–65 °C [49]. On heating the carnauba wax, one asymmetric exothermic peak at 82.3 ± 0.1 °C with an onset of 69.8 ± 0.1 °C was detected. The determined melting enthalpy was 190.1 ± 0.8 J/g. The continuous increase in the second heating curve before the two maxima was ascribed to the natural origin of the wax, which also contained some lower molecular weight compounds [84].

For the MCSs, the high-temperature endothermic peak shifted to 77.9 ± 0.3 °C (MCS_70:30_), 78.1 ± 0.3 °C (MCS_60:40_) and 78.4 ± 0.4 °C (MCS_50:50_), respectively. The shift between 0.8 and 1.3 °C was ascribed to the higher thermal conductivity of the MCSs compared to the pure wax. The lower endothermic peak was more symmetric and better separated for lower wax content samples. The onset of the endothermic heat absorption increased for the composite materials from 73.4 ± 0.2 °C to 76.4 ± 0.1 °C with the wax content decreasing and breaking off at 85.3 ± 0.3 °C (MCS_70:30_), 85.7 ± 0.5 °C (MCS_60:40_) and 86.2 ± 0.3 °C (MCS_50:50_). The reheating curves have a slightly different shape compared to the first cycles. The MCSs have lower heat of fusion than the pure wax, with values of 107.9 ± 1.7 J/g for MCS_70:30_, 95.0 ± 2.5 J/g for MCS_60:40_ and 69.5 ± 3.7 J/g for MCS_50:50_. As expected, the wax content (70, 60 and 50%) correlates with the percentage of heat accumulation relative to the pure wax, namely 57, 50 and 37%. The 17–26% lower values (compared to the theoretical ones with respect to the carnauba wax content) are related to the amorphization of the alkyl chains in the presence of the nanoparticles as well as to the quenching step in sample preparation.

Clays with a melting temperature of 1250–1300 °C do not participate in the accumulation of heat and serve primarily as a molding material as well as increasing the thermal conductivity of the composite material. To further investigate the effect of annealing, we subjected the MCS_50:50_ to annealing at 60 °C for a month and recorded the respective DSC curves (Supporting Information). The determined melting enthalpy was 84.5 ± 24.9 J/g. Considering the wide span of the measured enthalpies (Supporting Information), it seems that annealing results in enhanced inhomogeneity due to phase separation. However, this result is preliminary and requires a more detailed study.

*Mechanisms of thermal conductive transport.* In ordered solid materials, the thermal energy is transferred by electrons and lattice vibration and by molecular diffusion in the case of porous materials or materials with mobile components. For (conducting) metals, the main thermal transport mechanism involves mostly electrons, while for dielectric crystalline solids, it is achieved predominantly via lattice vibrations. According to the “phonon gas model”, a quantum of collective atomic vibrational energy is defined by a phonon, and the thermal transport in ordered non-conducting solids is presented as the diffusion of phonons driven by a temperature gradient [85]. Due to the lack of periodicity in amorphous solids, the “phonon gas model” no longer applies and is substituted with an ensemble of three different heat carries, i.e., propagons, diffusons, and locons (the latter being trapped in a local region do not significantly contribute to heat transport) [85,86]. While propagons (with low-frequency delocalized vibrational modes) still possess some periodicity and resemble (to some extent) phonons, the diffusions are delocalized random vibrational modes that are mostly responsible for the heat transport in amorphous solids [85]. The need to introduce propagons, etc., for thermal transport in glasses has been critically discussed recently [87]. It is also known that the inorganic additive limits the vibrational oscillations of organic molecules on a macroscopic level [56].

**Figure 7:**
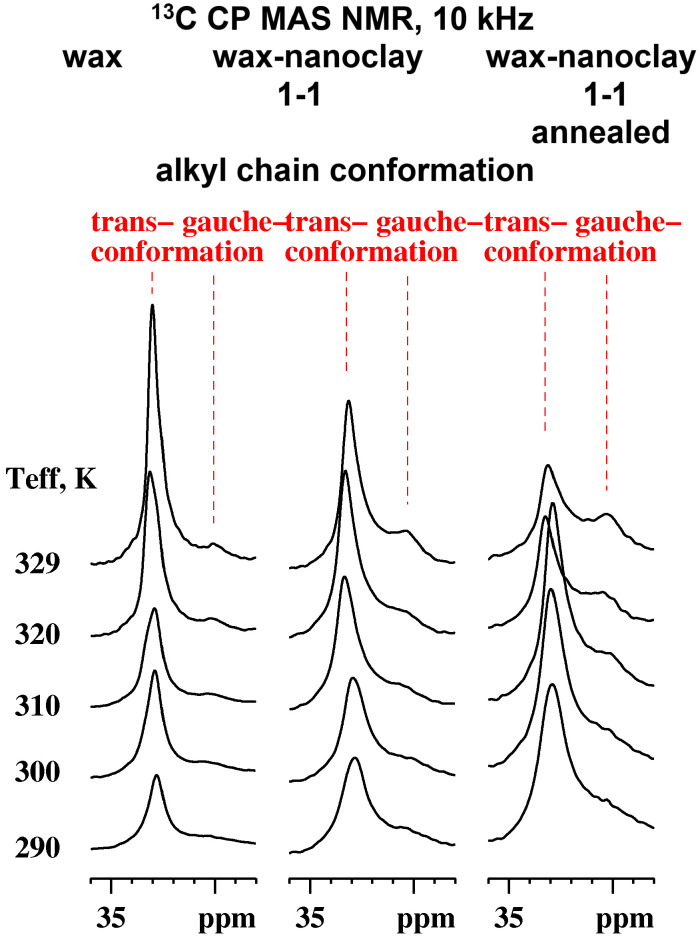
VT ^13^C CP NMR spectral region between 28 and 36 ppm of carnauba wax (**left**), pristine MCS_50:50_ (**middle**) and annealed MCS_50:50_ (**right**) recorded at 10 kHz of MAS and a contact time of 2 ms under proton decoupling with the effective sample temperature presented on the left.

The VT ^13^C CP NMR spectra (Figure 7) reveal an increase in the integral of the resonance related to the gauche conformation for the sake of the trans conformation increasing the temperature as well as in the row of wax, pristine MCS_50:50_, and annealed MCS_50:50_. This corresponds to the enhanced alkyl chain amorphization of the wax constituents in the composite materials (esp. after annealing) and suggests changes in the thermal conductive transport mechanisms compared to the pure wax as a function of temperature (below the melting point). The amorphization is not only related to the interface of nanoclay–carnauba wax components but also to the introduced (electrostatic) interactions between the polar functional groups (the wax alkoxyl− and ester COO^−^ groups) and the montmorillonite (charged) surface. Changes in π–π stacking during the preparation of composite materials and esp. during prolonged heat treatment also cannot be excluded. Thus, the contribution of the diffusions and (most probably) phonons compared to the lattice vibrations (for the remaining crystalline part of the wax constituents at high temperatures) in the thermal energy transport in the composite materials increases. Complementary thermal conductivity measurements should also be considered.

In the current contribution, we demonstrate that VT ^13^C CP NMR spectra are a convenient way to visualize the changes in the thermal conductive transport mechanism as a function of temperature following the changes in the alkyl chain conformation. This is a general approach related to any kind of system containing alkyl chains for latent heat storage. Also, VT solid-state NMR spectroscopy (which provided suitable NMR active nuclei and spectral differences for crystalline and amorphous domains) could be used to relate the observed spectral changes to changes in the thermal conductive transport mechanism.

## 4. Conclusions

Chemically stable PCMs were obtained in the form of mixtures of carnauba wax (ratios 50–70% (*w*/*w*)) and nanosized montmorillonite. In these MCSs, the nanoclay particles were inhomogeneously distributed. IR and multinuclear solid-state NMR spectroscopy confirmed that no structural changes took place during preparation. ^13^C VT ssNMR spectroscopy revealed amorphization of the alkyl chains of the wax components upon mixing with the montmorillonite at elevated temperatures. The reason for the macroscopic loss of order is the organic–inorganic interface and electrostatic interactions between the polar groups as well as interactions with the charged nanoparticle surface. We expect that this close interaction could enhance the mechanical properties of the MCSs.

The transformation of the alkyl chains conformation from trans to gauche, increasing the temperature, as well as in the mixtures, is related to changes in the thermal conduction transport mechanism from lattice vibration-based (higher crystallinity) to diffusion-based (higher amorphization). Using ^13^C CP MAS solid-state NMR spectroscopy is a general approach to following changes in the thermal conduction transport mechanism for wax-containing PCMs.

The DSC measurements reveal unequal contributions to heat accumulation comparing carnauba wax and nanoclay in the measured temperature range. As expected, the wax content in the MCSs correlated with the percentage of heat accumulation relative to the pure wax; however, was ca 17–26% lower than the theoretical values. The shifts in maxima in the DSC curves show that the heat transfer rates are somewhat higher for the mixtures with montmorillonite than for the pure wax.

The MCSs of this study are stable and consist of natural carnauba wax and montmorillonite. PCMs are very relevant for microclimate design and heat management in buildings, and petroleum waxes have suitable properties but suffer from a lack of shape stability on melting as fossil materials.

Carnauba wax has advantages over paraffin wax when it comes to sustainability and is available in large quantities and at a low price. The prepared MCSs are sustainable materials for energy storage and show promise for industrial applications. Life cycle and technoeconomica analyses and scale-up experiments are possibilities for future work.

## Data Availability

Data are contained within the article (and Supplementary Materials).

## Supplementary Materials

The following supporting information can be downloaded at: https://www.mdpi.com/article/10.3390/ma17091978/s1, Material characterization techniques–PXRD in the region of small diffraction angles of all materials (Figure S1) and PXRD of montmorillonite (Figure S2); FTIR spectra of the all materials (Figure S3); ^1^H, ^27^Al, ^27^Al cross-polarization and ^13^C cross-polarization NMR spectra as well as deconvoluted ^29^Si CP and ^27^Al NMR spectra (Figures S4–S10), together with the NMR parameters derived from the fitting procedure (Tables S1–S3); DSC curves for carnauba wax and the MCSs (Figures S11–S24) and tables with the derived DSC parameters (Tables S4–S8).
