# Supplementary material for: Sustainable Composite Materials Based on Carnauba Wax and Montmorillonite Nanoclay for Energy Storage
Source: Materials (Basel). 2024 Apr 24;17(9):1978. doi: 10.3390/ma17091978 (PMC11084883; doi:10.3390/ma17091978)
Supplement: Supplementary file 1 [file materials-17-01978-s001.zip › materials-2916273-SI.pdf]

Supporting Information for

Sustainable Composite Materials based on Carnauba Wax and Montmorillonite Nanoclay for Energy Storage

Serhii Brychka[a,b,c]*, Alla Brychka[a,b,d], Niklas Hedin[b], Mihail Mondeshki[a]*

[a]*Department Chemie, Johannes-Gutenberg-Universität, Duesbergweg 10-14, 55128 Mainz, Germany*

[b]*Department of Materials and Environmental Chemistry, Stockholm University, SE-106 91 Stockholm, Sweden*

[c]*The Gas Institute of the National Academy of Sciences of Ukraine, 39, Dehtyarivska Str., 03113 Kyiv, Ukraine*

[d]*Chuiko Institute of Surface Chemistry of National Academy of Sciences, 17 General Naumov Street, Kyiv 03164, Ukraine*

* *e-mail: serg_1971@ukr.net; mondeshk@uni-mainz.de*

XRD spectra

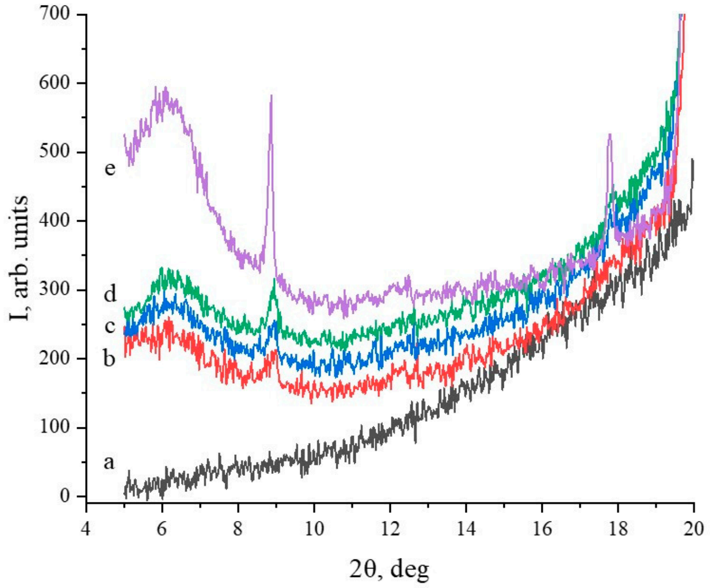

Figure S1. XRD patterns of mixtures of wax (a) with montmorillonite clay (w/w): (b) $MCS_{70:30}$, (c) $MCS_{60:40}$, (d) $MCS_{50:50}$, and the pure montmorillonite clay (e).

The diffraction peak at 2θ = 26.64 degrees corresponds to quartz while the one at 2θ = 20.84 degrees to feldspar (Figure S2). The diffraction peak (001) is observed in the region of small diffraction angles (2θ = 4-9 degrees). For montmorillonite in the nanocomposites, compared to natural montmorillonite (2θ = 8.86 degrees), no shift of the peak maximum (001) is observed (Figure S1).

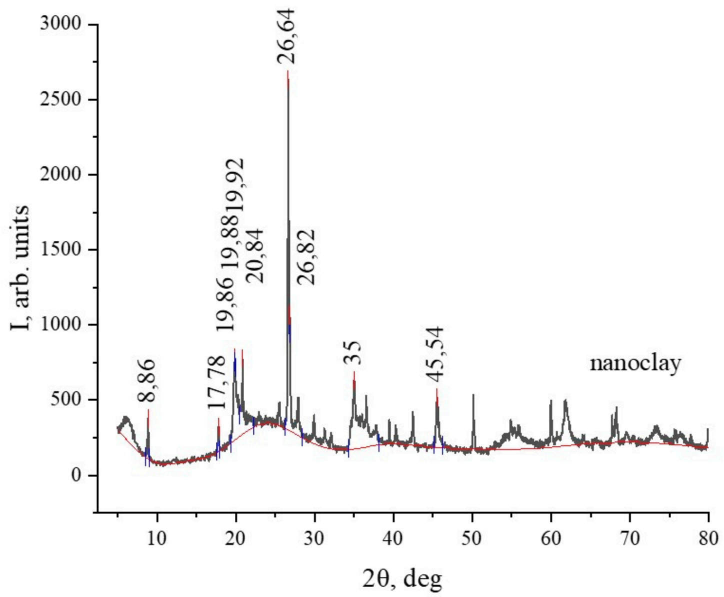

Figure S2. XRD patterns of the montmorillonite clay.

FTIR spectra

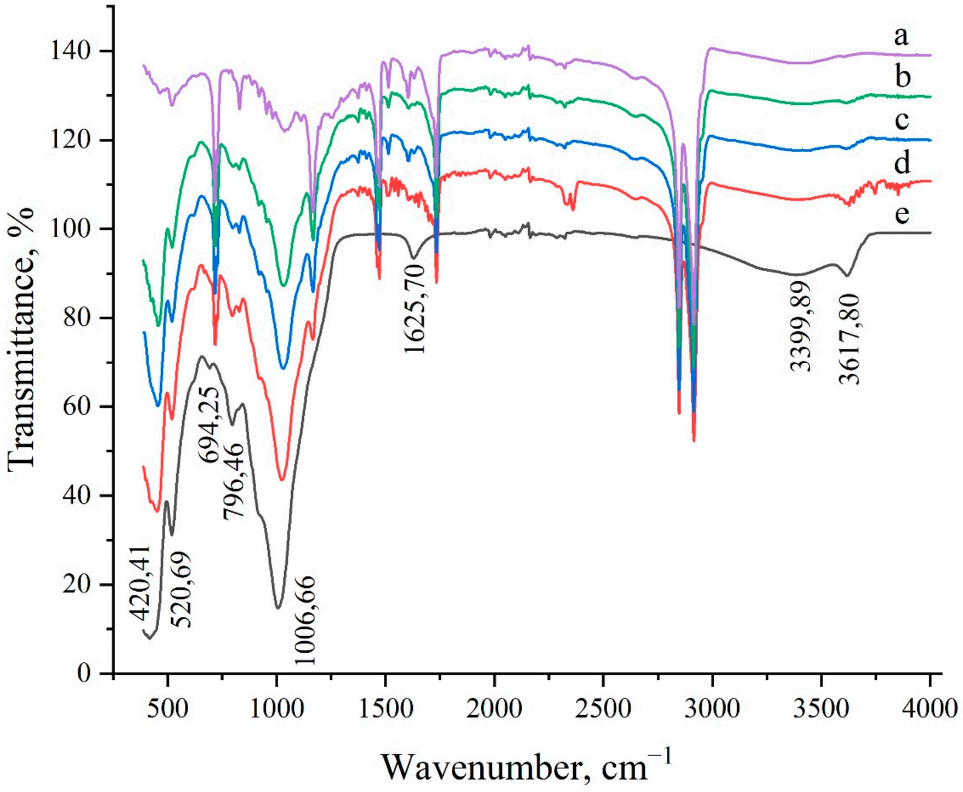

Figure S3. IR spectra of (a) pure wax, and its mixtures with the montmorillonite clay (w/w): (b) $MCS_{70:30}$, (c) $MCS_{60:40}$, (d) $MCS_{50:50}$, and the pure montmorillonite clay (e).

NMR spectra

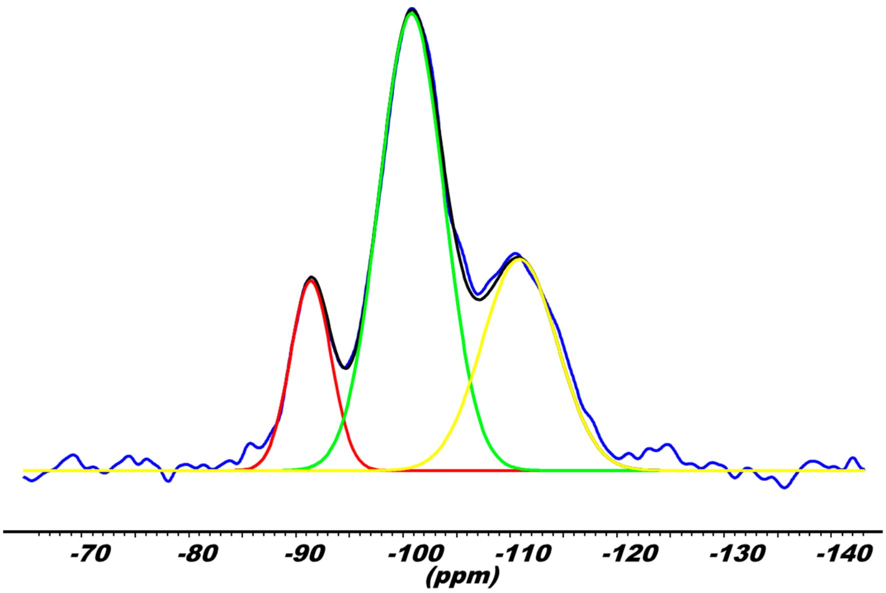

Figure S4. Deconvoluted ^{29}Si CP NMR spectrum of the pure montmorillonite nanoclay with the derived parameters from the fit included in Table S1

Table S1. NMR parameter derived from the deconvoluted spectrum

Peak position, ppm	Intensity, a.u.	FWHM, Hz	Gauss/Lorentz ratio	Integral, a.u.
-91.4348	119477032	354.00	1.0	3.473982e+010
-100.8384	288106432	567.63	1.0	1.343254e+011
-110.9246	133179568	654.69	1.0	7.161651e+010

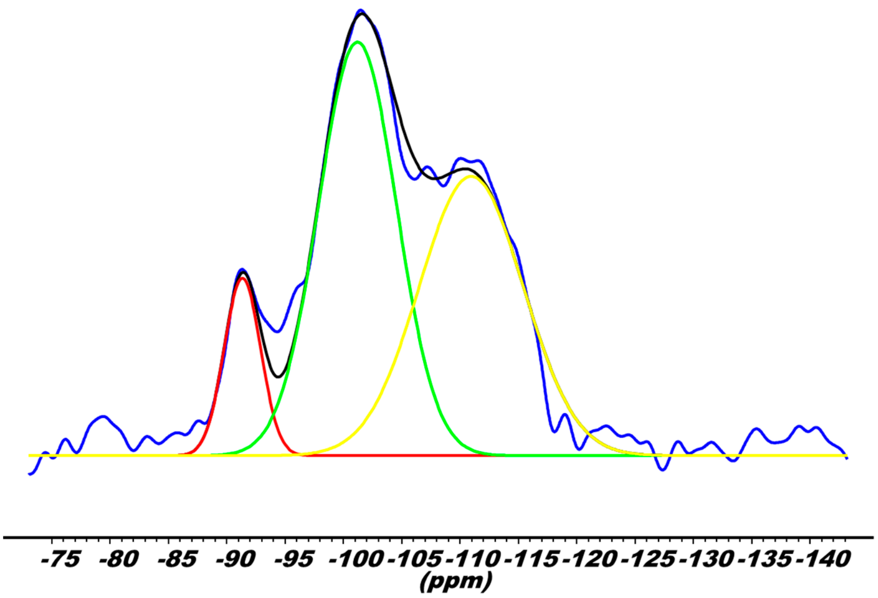

Figure S5. Deconvoluted ^{29}Si CP NMR spectrum of the $MCS_{50:50}$ sample with the derived parameters from the fit included in Table S2

Table S2. NMR parameter derived from the fit of the deconvoluted spectrum

Peak position, ppm	Intensity, a.u.	FWHM, Hz	Gauss/Lorentz ratio	Integral, a.u.
-91.3387	129711464	290.66	1.0	3.096731e+010
-101.1943	301206176	625.61	1.0	1.547774e+011
-110.9422	203592992	833.13	1.0	1.393206e+011

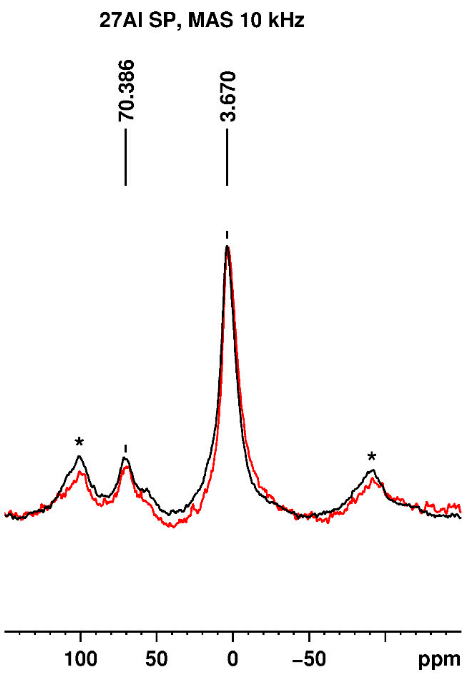

Figure S6 ^{27}Al single pulse (SP) excitation NMR spectra of montmorillonite (black) and $MCS_{50:50}$ (red) recorded at 10 kHz MAS. The spectra are scaled to equal intensity. The asterisks denote spinning sidebands.

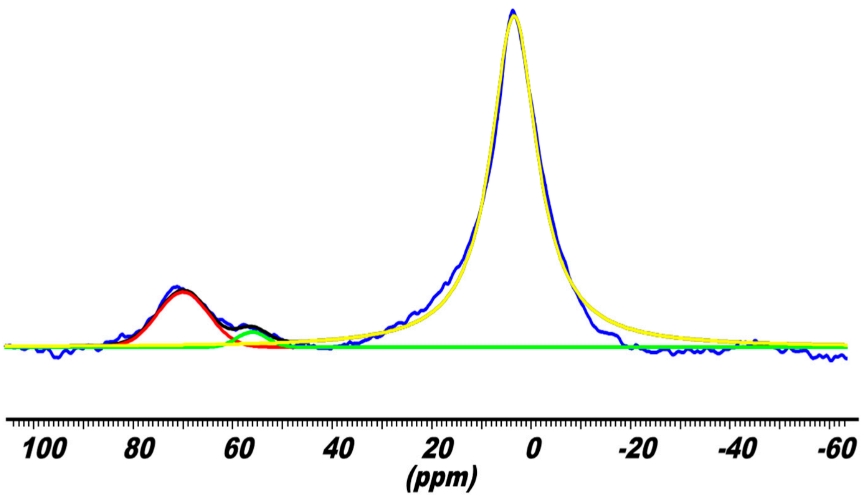

Figure S7. Deconvoluted ^{27}Al SP NMR spectrum of montmorillonite with the derived parameters from the fit included in Table S3

Table S3. NMR parameter derived from the fit of the deconvoluted ^{27}Al SP NMR spectrum

Peak position, ppm	Intensity, a.u.	FWHM, Hz	Gauss/Lorentz ratio	Integral, a.u.
69.9598	9011382	1261.89	1.0	9.340127e+009
56.0585	2581234	748.55	1.0	1.587041e+009
3.3853	54658712	1181.92	0.0	6.650393e+010

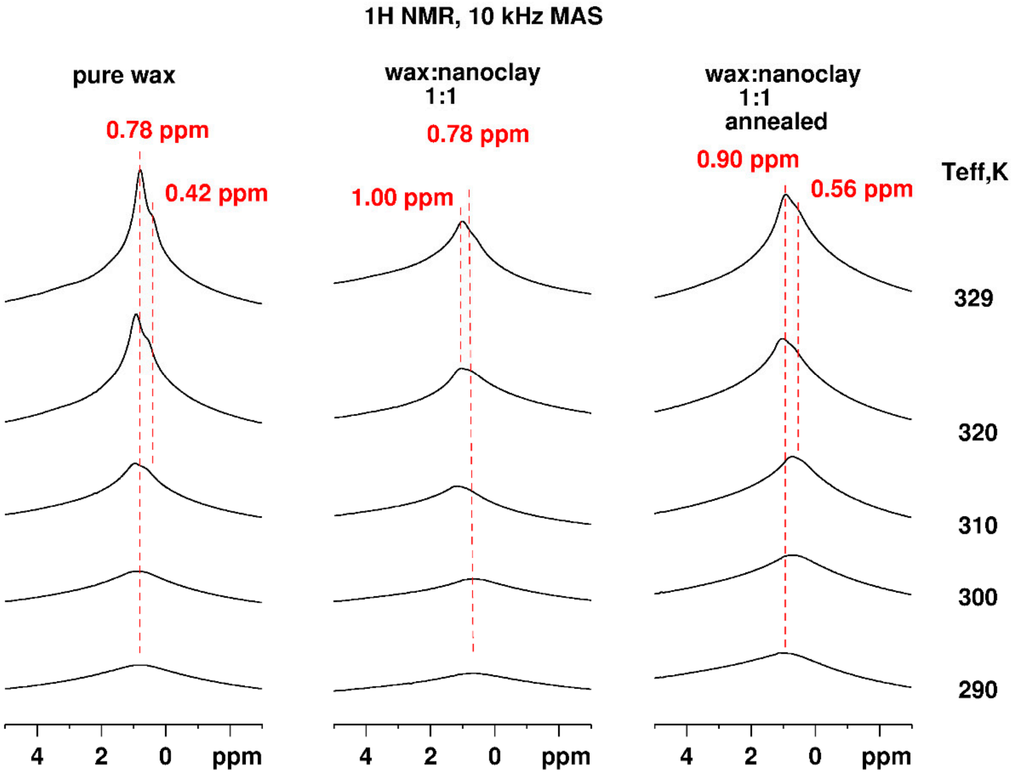

Figure S8. VT ^{1}H NMR spectra of the pure carnauba wax, the pristine $MCS_{50:50}$ and the annealed $MCS_{50:50}$ at 60°C at 290-329 K.

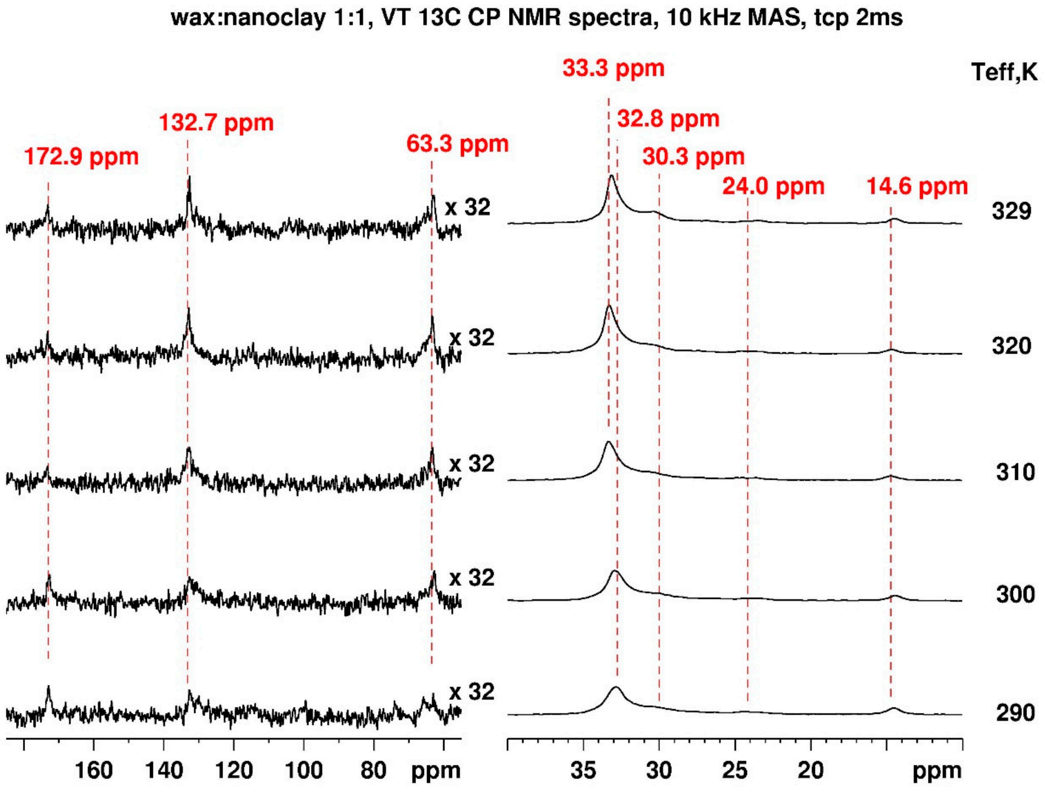

Figure S9. VT ^{13}C CP NMR spectra of the pristine $MCS_{50:50}$ recorded at 10kHz MAS and contact time 2 ms under proton decoupling with the effective sample temperature presented on the right.

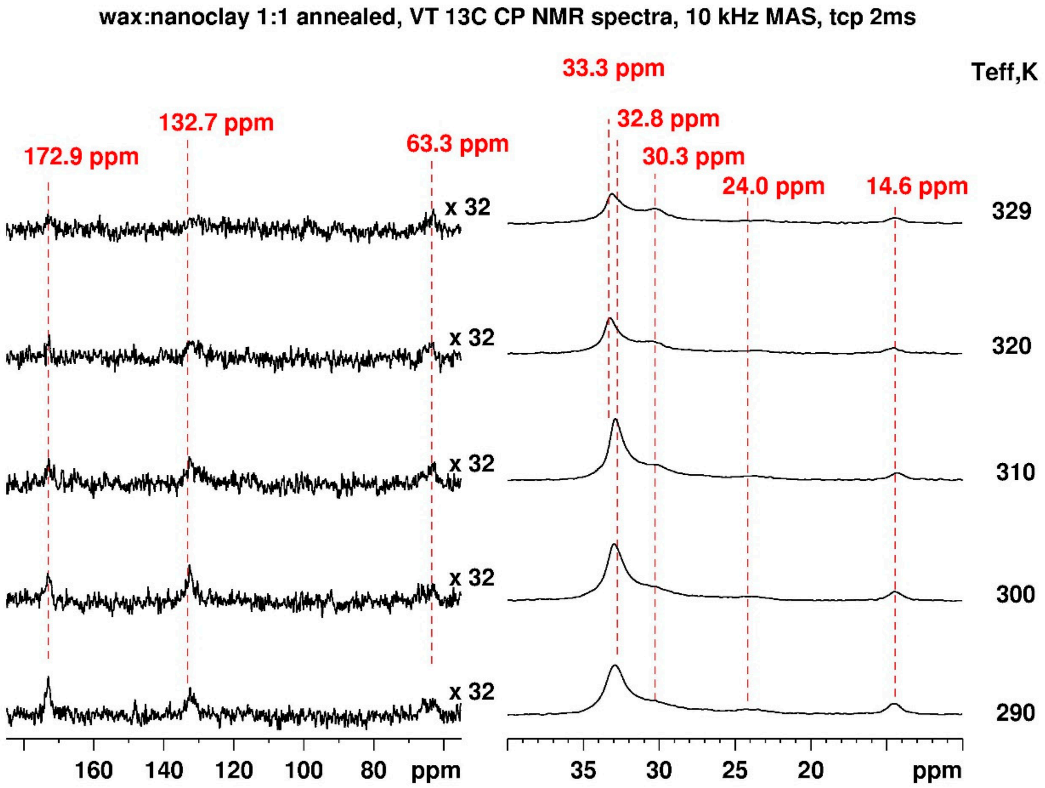

Figure S10. VT ^{13}C CP NMR spectra of annealed $MCS_{50:50}$ recorded at 10kHz MAS and contact time 2 ms under proton decoupling with the effective sample temperature presented on the right.

DSC thermograms

Tables with the characteristic values from DSC thermograms for the second melting of pure wax, $MCS_{70:30}$, $MCS_{60:40}$, $MCS_{50:50}$ and annealed $MCS_{50:50}$

Table S4. Characteristic DSCs value extracted from the second melting of pure wax (Wax : Montmorillonite 100 : 0) from the thermograms S11 and S12

	Normalized Enthalpy, J/g	Onset, °C	Peak temp., °C	Endset, °C
1	190.9	69.8	82.3	85.7
2	189.3	69.7	82.2	85.8
average	190.1 ± 0.8	69.8 ± 0.1	82.3 ± 0.1	85.8 ± 0.1

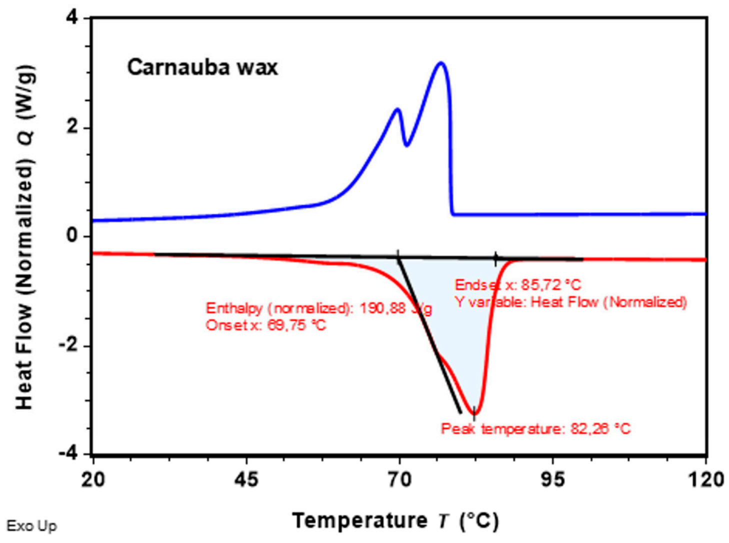

Figure S11. DSC thermogram of carnauba wax (first measurement)

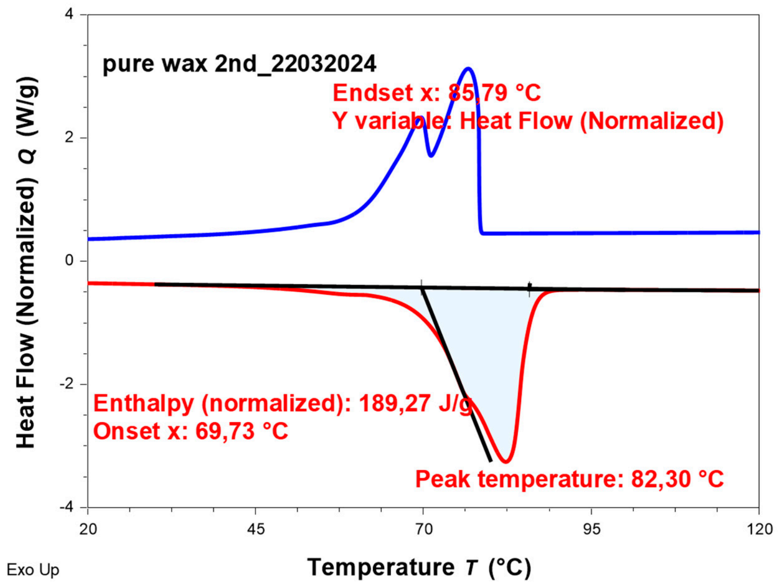

Figure S12. DSC thermogram of carnauba wax (second measurement)

Table S5. Characteristic DSCs value extracted from the second melting of $MCS_{70:30}$ (Wax : Montmorillonite 70 : 30) from the thermograms presented on Figures S13, S14 and S15

	Normalized Enthalpy, J/g	Onset, °C	Peak temp., °C	Endset, °C
1	106.7	73.6	82.6	85.0
2	107.3	73.4	82.9	85.3
3	109.6	73.2	83.0	85.6
average	107.9 ± 1.7	73.4 ± 0.2	82.8 ± 0.2	85.3 ± 0.3

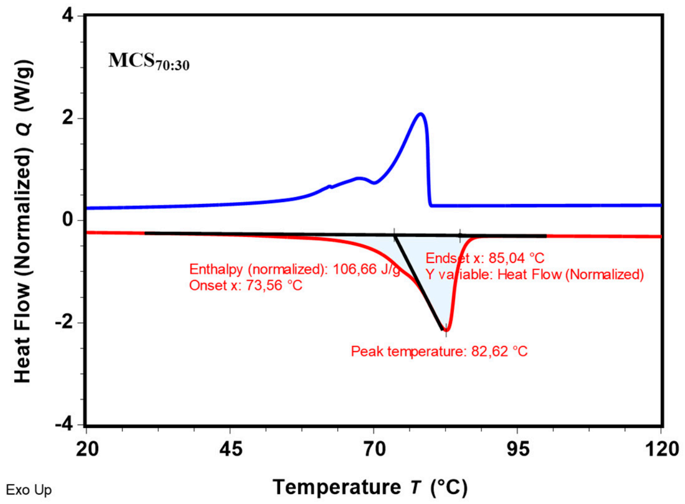

Figure S13. DSC thermogram of $MCS_{70:30}$ (first measurement)

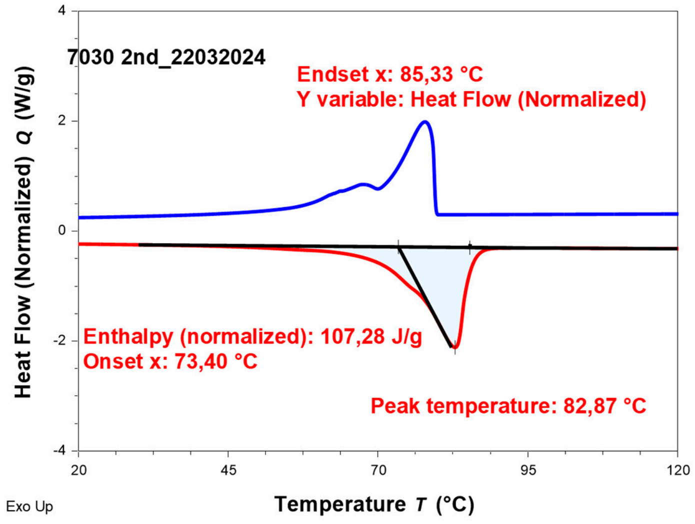

Figure S14. DSC thermogram of $MCS_{70:30}$ (second measurement)

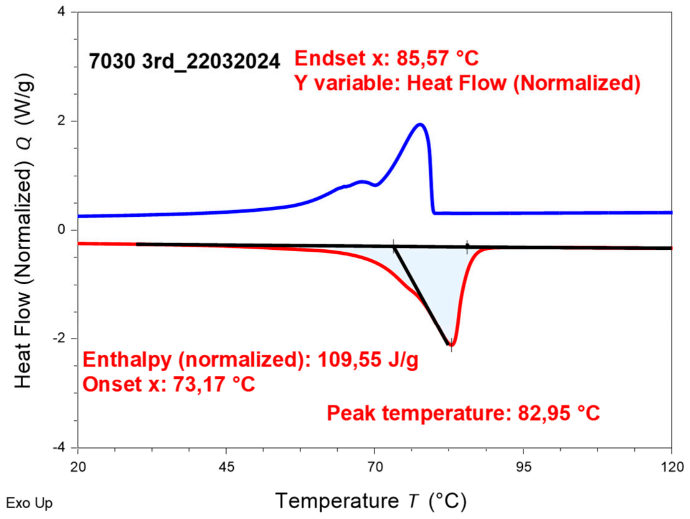

Figure S15. DSC thermogram of $MCS_{70:30}$ (third measurement)

Table S6. Characteristic DSCs value extracted from the second melting of $MCS_{60:40}$ (Wax : Montmorillonite 60 : 40) from the thermograms presented on Figures S16, S17 and S18

	Normalized Enthalpy, J/g	Onset, °C	Peak temp., °C	Endset, °C
1	92.5	74.6	82.9	85.2
2	95.7	74.3	83.4	86.0
3	96.7	74.2	83.3	85.9
average	95.0 ± 2.5	74.4 ± 0.2	83.2 ± 0.3	85.7 ± 0.5

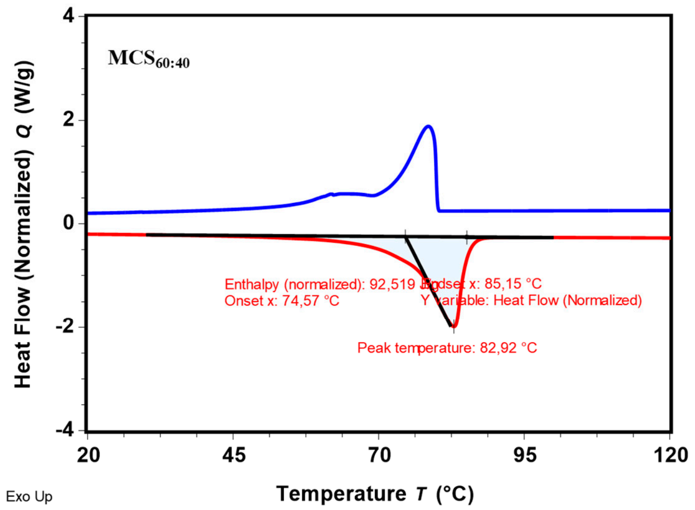

Figure S16. DSC thermogram of $MCS_{60:40}$ (first measurement)

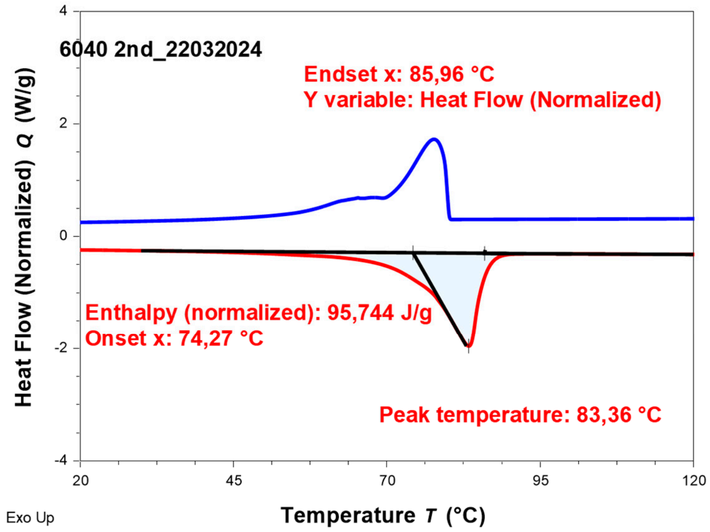

Figure S17. DSC thermogram of $MCS_{60:40}$ (second measurement)

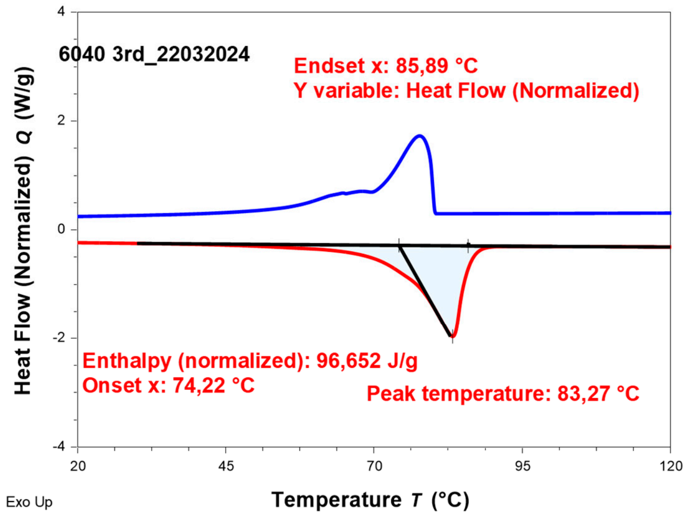

Figure S18. DSC thermogram of $MCS_{60:40}$ (third measurement)

Table S7. Characteristic DSCs value extracted from the second melting of $MCS_{50:50}$ (Wax : Montmorillonite 50 : 50) from the thermograms presented on Figures S19, S20 and S21

	Normalized Enthalpy, J/g	Onset, °C	Peak temp., °C	Endset, °C
1	65.8	76.5	83.8	86.5
2	71.7	76.4	83.5	86.1
3	70.9	76.4	83.5	85.9
average	69.5 ± 3.7	76.4 ± 0.1	83.6 ± 0.2	86.2 ± 0.3

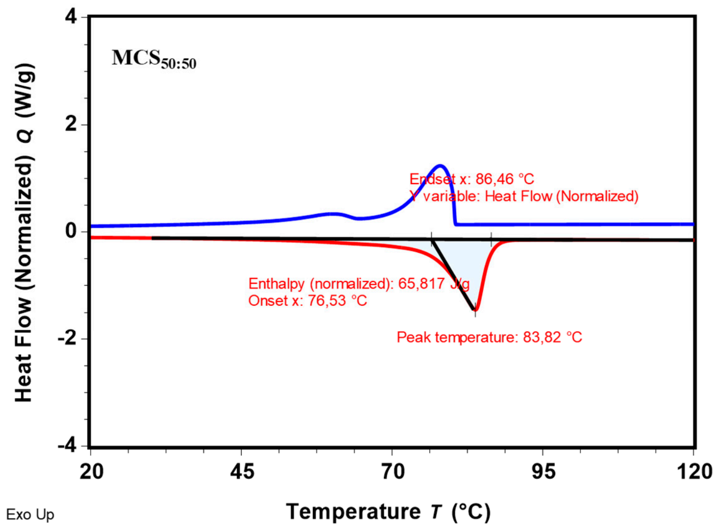

Figure S19. DSC thermogram of $MCS_{50:50}$ (first measurement)

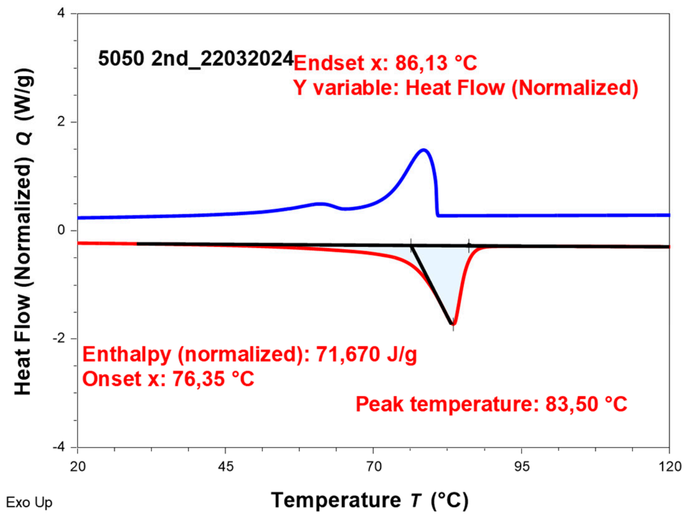

Figure S20. DSC thermogram of $MCS_{50:50}$ (second measurement)

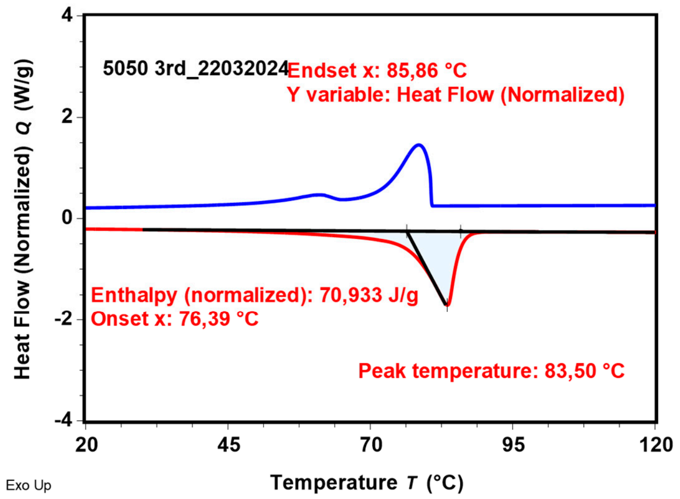

Figure S21. DSC thermogram of $MCS_{50:50}$ (third measurement)

Table S8. Characteristic DSCs value extracted from the second melting of annealed $MCS_{50:50}$ (Wax : Montmorillonite 50 : 50) from the thermograms presented on Figures S22, S23 and S24

	Normalized Enthalpy, J/g	Onset, °C	Peak temp., °C	Endset, °C
1	109.4	75.3	83.2	86.8
2	74.6	76.8	84.1	87.8
3	69.4	77.1	84.2	87.2
average	84.5 ± 24.9	76.4 ± 1.1	83.8 ± 0.6	87.3 ± 0.5

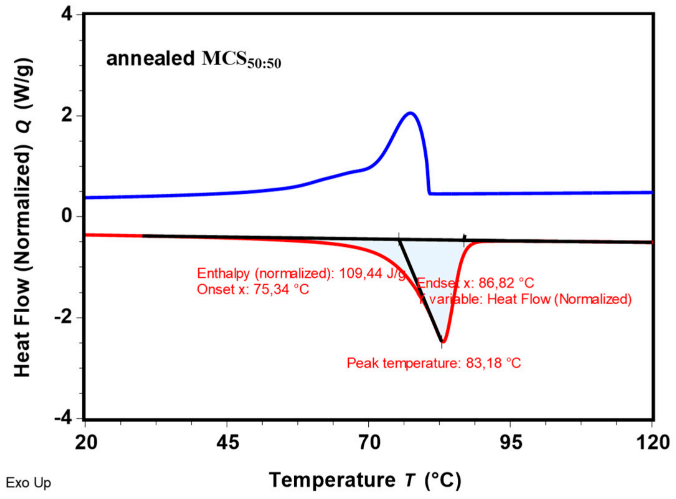

Figure S22. DSC thermogram of annealed $MCS_{50:50}$ (first measurement)

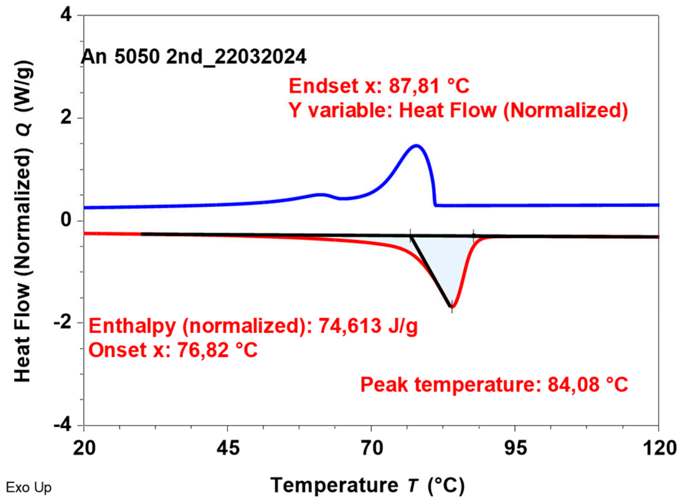

Figure S23. DSC thermogram of annealed $MCS_{50:50}$ (second measurement)

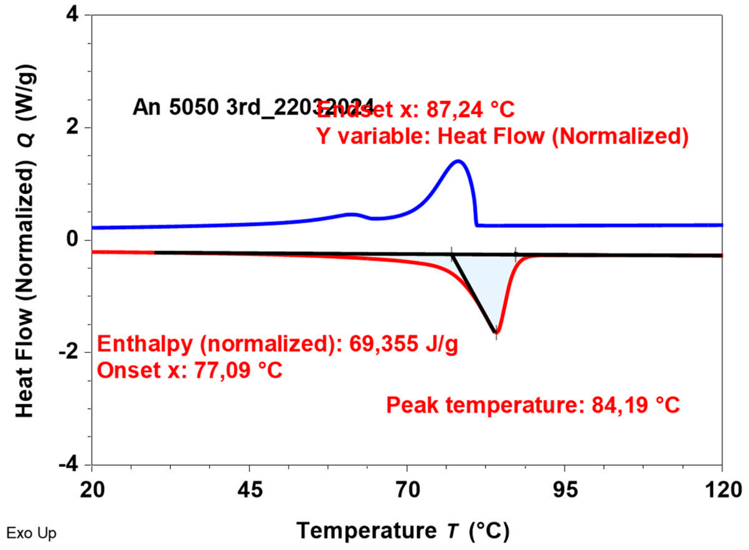

Figure S24. DSC thermogram of annealed $MCS_{50:50}$ (third measurement)
